# Radiotherapy Plus Concurrent or Sequential Temozolomide for Glioblastoma in the Elderly: A Meta-Analysis

**DOI:** 10.1371/journal.pone.0074242

**Published:** 2013-09-24

**Authors:** An-an Yin, Lu-hua Zhang, Jin-xiang Cheng, Yu Dong, Bo-lin Liu, Ning Han, Xiang Zhang

**Affiliations:** 1 Department of Neurosurgery, Xijing Institute of Clinical Neuroscience, Xijing Hospital, Fourth Military Medical University, Xi’an, Shaanxi Province, PR China; 2 Department of Prosthodontics, School of Stomatology, Fourth Military Medical University, Xi’an, Shaanxi Province, PR China; Istituto di Ricerche Farmacologiche Mario Negri, Italy

## Abstract

**Background:**

Many physicians are reluctant to treat elderly glioblastoma (GBM) patients as aggressively as younger patients, which is not evidence based due to the absence of validated data from primary studies**.** We conducted a meta-analysis to provide valid evidence for the use of the aggressive combination of radiotherapy (RT) and temozolomide (TMZ) in elderly GBM patients.

**Methods:**

A systematic literature search was conducted using the PubMed, EMBASE and Cochrane databases. Studies comparing combined RT/TMZ with RT alone in elderly patients (≥65 years) with newly diagnosed GBM were eligible for inclusion.

**Results:**

No eligible randomized trials were identified. Alternatively, a meta-analysis of nonrandomized studies (NRSs) was performed, with 16 studies eligible for overall survival (OS) analysis and nine for progression-free survival (PFS) analysis. Combined RT/TMZ was shown to reduce the risk of death and progression in elderly GBM patients compared with RT alone (OS hazard ratio [HR] 0.59, 95% confidence interval [CI] 0.48–0.72; PFS: HR 0.58, 95% CI 0.41–0.84). Evaluable patients were reported to tolerate combined treatment but certain toxicities, and especially hematological toxicities, were more frequently observed. Limited data on O6-methylguanine-DNA methyltransferase (MGMT) promoter status and quality of life were reported.

**Conclusion:**

The meta-analysis of NRSs provided level 2a evidence (Oxford Centre for Evidence-Based Medicine) that combined RT/TMZ conferred a clear survival benefit on a selection of elderly GBM patients who had a favorable prognosis (e.g., extensive resection, favorable KPS). Toxicities were more frequent but acceptable. Future randomized trials are warranted to justify a definitive conclusion.

## Introduction

Glioblastoma (GBM) is the most frequent and most devastating brain malignancy. The typical survival range for GBM is 12–15 months, despite the aggressive use of surgery, radiotherapy (RT) and chemotherapy [1]. The prognosis of elderly patients is even poorer, and population-based survival is only 4–6 months [2]–[3].

The optimal treatment of GBM in the elderly remains highly controversial. Many physicians are reluctant to treat elderly patients as aggressively as younger patients, citing concerns about overall poor physical condition, the common presence of comorbidity and decreased tolerance to effective therapies [4]. Those conservative treatment decisions, however, are not evidence based due to the absence of validated data from primary studies [4]. The European Organization for Research on Treatment Cancer (EORTC) 26981 trial set the standard of treatment as RT with concurrent and adjuvant temozolomide (TMZ) for adult patients with GBM. Patients older than 70 years, however, were excluded from the trial, and the survival of patients older than 65 years did not show any significant difference between the groups [5]–[6]. This subset analysis of a few patients was statistically underpowered and did not justify any definitive conclusion. Overall, the aggressive combination of RT and TMZ has not yet been justified in elderly GBM patients. Recently, an increased number of elderly patients have been enrolled in clinical studies in which, the potential benefits of combined treatment were highlighted. The aim of our study was to systematically review clinical data from those studies and to provide valid evidence for the use of the aggressive RT/TMZ combination in elderly patients with newly diagnosed GBM.

## Methods

### Eligibility Criteria

#### Types of studies

Randomized and non-randomized studies (NRSs).

#### Type of participants

Patients aged 65 years and over with newly diagnosed, histologically confirmed GBM.

#### Types of interventions

Studies comparing postoperative RT plus concomitant or sequential TMZ with RT alone were eligible. Different schedules or delivery modalities for TMZ and RT were all included.

#### Types of outcome measures

Outcomes of interest included 1) overall survival (OS) defined as the time interval from the date of diagnosis or treatment to the date of death or last follow-up; 2) progression-free survival (PFS) defined as the interval from the date of diagnosis or treatment to the date of progression, which was defined using the Macdonald criteria [7] or to the date of death or last follow-up without progression; 3) adverse events, classified according to WHO criteria or National Cancer Institute Common Terminology Criteria (NCI-CTC); and 4) health-related quality of life (HRQOL), which was assessed using a validated scale.

### Literature Search

A literature search was performed using the PubMed, Cochrane Library and EMBASE bibliographic databases and the search strings “glioma”, “glioblastoma”, “malignant glioma”, “high grade glioma”, “elderly”, “advanced age”, “older patients”, “radiotherapy”, “chemotherapy”, “chemoradiation”, “temozolamide”, “temozolomide” and “Temodar”, and no restriction was applied to the language or publication date. Related articles identified using electronic databases and reference lists from relevant articles were also reviewed.

### Studies Selection and Data Extraction

Study selection was independently conducted by two reviewers (YAA and ZLH), where disputes were resolved by discussion. The reviewers were not blinded to the study identity (e.g., authors, contact address, sources) during eligibility assessment. The newest publication of an identical cohort was collected.

An extraction sheet was developed according to the Cochrane Non-Randomised Studies Methods Group [8] and, was tested in several studies and well customized to the topic of the current study. Data from eligible studies were extracted, as follows: 1) study identity (e.g., authors, publication data, contact address), 2) study design feature (e.g., prospective or retrospective), 3) participants (e.g., baseline characteristics, inclusion and exclusion criteria, losses to follow-up), 4) study intervention (e.g., schedules, delivery modalities, comparison intervention), and 5) study outcome (e.g., OS, PFS, adverse events, HRQOL scores).

### Assessment of Risk of Bias in Eligible Studies

The risk of bias in each study was assessed by two independent reviewers (YAA and CJX) using either the Cochrane Collaboration’s standard evaluation for randomized trials [8] or a modified Newcastle Ottawa Scale (NOS) for NRSs [9]. Within the modified NOS, to evaluate the comparability of baseline characteristics between the groups receiving different interventions, we focused on the following important prognostic variables: age, surgery, KPS, tumor number or location, neurological status, promoter status of the O6-methylguanine-DNA methyltransferase (MGMT) gene and comorbidities. The judgment criteria for the modified evaluation are explicitly described in Table S1.

### Statistical Analysis

Time-to-event data (e.g., OS, PFS) were analyzed using hazard ratios (HR). If the HR was not reported, the value was estimated using the calculation methods described by Tierney et al [10]. For proportions (e.g., the percentage of patients who experienced at least one adverse event of interest, survival rate), point estimates and 95% confidence intervals (CIs) were computed using the logit transformation formula. [11] The inverse-variance approach was implemented using either fixed- or random-effect models, which are based on the heterogeneity of included studies.

Heterogeneity was tested using the Chi^2^ test and I^2^ statistic (the percentage of the total variation in the overall results that is due to heterogeneity rather than chance), with P*_heterogeneity_*<0.1 or I^2^>50% considered to be statistically significant.

Publication bias was assessed by visual examination of the funnel plots and by analytic methods (e.g., Egger’s test [12], Duval and Tweedie’s *trim and fill* [13]).

Given a greater likelihood of substantial variations within NRSs [8], several strategies for addressing heterogeneity were implemented, as follows: 1) subgroup analyses of different study design features and different schedules of RT or TMZ were undertaken, and 2) a meta-regression analysis was used to assess variations in pooled estimates by potential confounding variables.

Sensitivity analyses were performed by considering the risk of bias of the studies and variables that were not closely relevant to our topic.

All analyses were performed using Review Manager V5.2 (The Cochrane Collaboration, Oxford, UK), Comprehensive Meta-Analysis V2.0 (Biostat, Englewood, NJ, USA) and R V2.15.3 (R Foundation for Statistical Computing, Vienna, Austria).

## Results

### Characteristics of Included Studies

Using the pre-specified eligibility criteria, we only identified one ongoing phase 3 trial (the NCIC CE.6/EORTC 26062/22061 trial) [14] comparing short course RT plus concurrent and adjuvant TMZ with short course RT alone in older GBM patients, which however insufficient data could currently be obtained.

**Figure 1:**
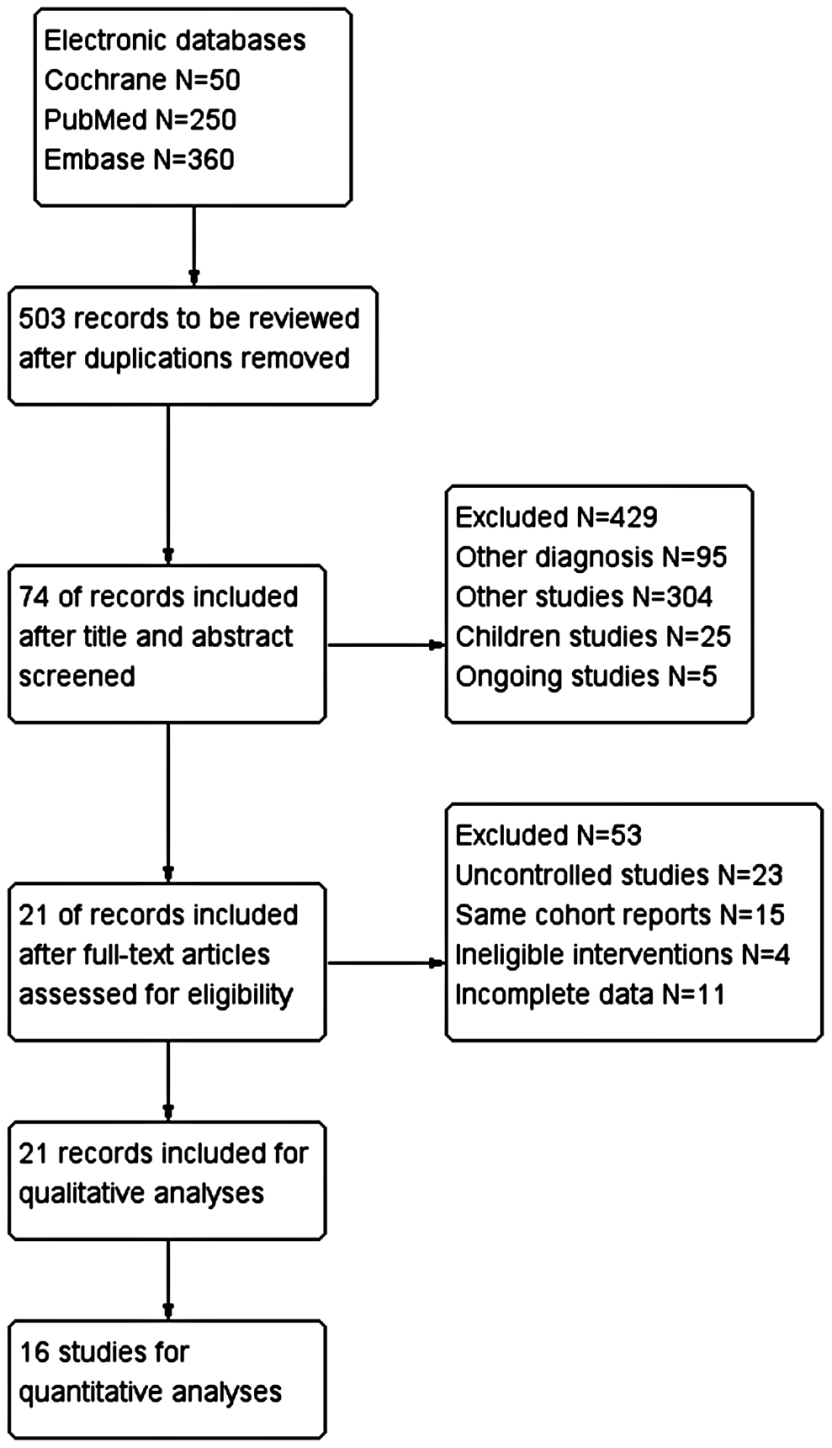
Flow diagram of study selection.

The literature search also identified 21 NRSs deemed to meet the trial eligibility criteria [5], [15]–[34], so a meta-analysis of NRSs was performed (Fig. 1). Of the NRSs, five had a prospective design [5], [16], [25], [28], [32], 16 had a retrospective design [15], [17]–[24], [26]–[27], [29]–[31], [33]–[34], and five were published in abstract [15], [17], [31], [33]–[34]. The characteristics of the included NRSs are summarized in Table 1.

**Table 1:** Characteristics of included studies.

Study reference	Cutoff age	Period ofdiagnosis	No. ofpatients	Therapy	MedianAge (yrs)	Median KPS(range)	Male%	Sx%	CR%	Median OS (mths,95% CI)	Median PFS(mths, 95% CI)	Patients at leastone G3–4 HAE
**Combination of standard RT and TMZ**												
Ackerl 2012 [16]	≥65	2003–2009	12 frail	RT	–	–	–	–	–	3 (nr)	–	1 in 12^c^
			23 fit	RT +concurrent TMZ	–	–	–	–	–	21 (nr)	–	2 in 23
Brandes 2003 [17]	≥65	1993–2000	24	RT	70	72.5 (60–90)	–	–	42	11.2 (9.4–13.4)	5.3 (4.8-7)	–
			23	RT +sequential TMZ	68	77 (60–90)	–	–	44	14.9 (13.4–24.4)	10.7 (8.4–16.5)	–
Dirier 2010 [18]	≥60	–	183	RT	–	70 (30–90)^b^	59^b^	81^b^	41^b^	8 (nr)	–	–
			122	RT +either TMZ						12 (nr)	–	13 in 122
Ewelt 2011 [19]	≥65	2002–2007	37	RT	70.6	70 (nr)	54	51#	22#	4.4 (nr)	3.2 (nr)	–
			35	RT +either TMZ	68.5	80 (nr)	51	89	40	15 (nr)	6.4 (nr)	–
Kimple 2010 [20]	≥70	2002–2007	4	RT	75.5	70 (40–80)	50	100	25	28.1 (nr)	–	–
			14	RT +either TMZ	74	80 (60–90)	64	57	43	50.4 (nr)	–	1 in 10
Kushnir 2011 [21]	≥65	1996–2007	8	RT	–	–	–	100	–	9.5 (nr)	–	–
			27	RT +unclear TMZ	–	–	–	100	–	12.1 (nr)	–	–
Mangiola 2006 [22]	≥65	1999–2003	11	RT	–	–	–	–	–	7 (nr)	–	–
			6	RT +sequential TMZ	–	–	–	–	–	15 (nr)	–	–
Niyazi 2012 [23]	≥70	2002–2009	25	RT	75	80 (70–80)	52	32	–	10.5 (8.4–12.6)	–	–
			18	RT +concurrent TMZ	76	70 (70–80)	56	11	–	6.4 (4.3–8.5)	–	–
Piccirilli 2006 [24]	≥80	2000–2004	10	RT	85#	70 (60–80)	–	80	80	16 (nr)	10 (nr)	–
			6	RT +concurrent TMZ	82	80 (70–100)	–	100	100	21 (nr)	12 (nr)	1 in 6
Sijben 2008 [25]	≥65	2004–2007	20	RT	70.5#	70 (50–90)#	75	45#	–	5.1 (1.5–14.2)	4.1 (1.5–14.2)	–
			19	RT +concurrent TMZ	67	80 (60–90)	53	89	–	8.5 (2–24.7)	6 (1.6–24.7)	3 in 19
Stummer 2011 [26]	≥60	2005–2007	64	RT or upfront TMZ or Sx	66	90 (70–100)#	70	100	–	11.2 (7.4–14.1)	–	–
			66	RT +concurrent TMZ	68	90 (70–100)	65	100	–	16.3 (12–17.2)	–	–
Stupp 2009 [5]	≥65	2000–2002	83^b^	RT	–	–	–	–	–	–	–	–
				RT +concurrent TMZ	–	–	–	–	–	–	–	–
Tanaka 2012 [27]	≥65	2003–2008	23	RT	75.6a,#	73.5 (60–90)a,#	57	52	–	4.5 (nr)	–	–
			41	RT +concurrent TMZ*	72.5^a^	79.8 (60–90)^a^	56	59	–	11.5 (nr)	–	5 in 42^d^
**Combination of short course RT and TMZ**												
Cao 2012 [28]	≥60	2000–2009	55	RT	70^a^	70 (30–90)	65	62#	33	9.3 (5.9–11.8)	–	2 in 24^e^
			57	RT +concurrent TMZ	70^a^	80 (30–100)	65	42	30	6.9 (4.5–8.6)	–	5 in 57
Muni 2010 [29]	≥70∧	2002–2006	23	RT	66	70 (50–90)	57	74	–	7.3 (nr)	4.4 (nr)	–
			22	RT +sequential TMZ	67	70 (50–90)	55	68	–	9.4 (nr)	5.5 (nr)	8 in 22
**Combination of mixed or unclear course RT and TMZ**												
Abhinav 2013 [30]	≥65	2007–2009	37	RT or Sx	73^b^	2 (0–4)b, f	64^b^	56^b^	43^b^	4.8 (nr)	–	–
			22	RT +concurrent TMZ						10.5 (nr)	–	–
Barker 2012 [31]	≥65	1987–2008	176	RT	71	80 (40–100)	–	83	31	<70yrs: 12 (10–15) ≥71yrs: 10 (8–11)	–	–
			115	RT +concurrent TMZ	71	80 (40–100)	–	83	45	<70yrs: 21 (13–24) ≥71yrs: 13 (8–17)	–	10 in 84
Caroli 2011 [32]	≥70	2005–2010	9	RT	73^b^	nr (≥70)^b^	65^b^	88^b^	75^b^	7.8 (nr)	–	–
			45	RT +concurrent TMZ						11.6 (nr)	–	–
Reifenberger 2011 [33]	≥70	2004–2010	61	RT	–	–	–	–	–	8.7 (7–10.4)	5 (4.4–5.6)	–
			91	RT +unclear TMZ	–	–	–	–	–	12.3 (11.2–13.4)	7.2 (6.3–8)	–
Sharp 2011 [34]	≥70	2004–2010	51^b^	RT	75^b^	–	–	–	–	5 (nr)	–	–
				RT +unclear TMZ						11 (nr)	–	–
Smith 2012 [35]	≥65	2006–2010	28	RT	–	–	–	–	–	6.9 (4.4–9.5)	–	–
			69	RT +unclear TMZ	–	–	–	–	–	13.1 (11.6–14.5)	–	–

RT = radiotherapy; TMZ = temozolomide; either TMZ = either sequential or concurrent TMZ; Sx = surgery; CR = complete resection; PFS = progression free survival; OS = overall survival; G3–4 HAE = grade 3–4 hematological adverse event; CI = confidential interval; yrs = years; mths = months; nr = not reported.

a mean but not median.

b the whole cohort.

c one hematological event probably due to the use of dexamethasone.

d additional one patients received upfront TMZ.

e patients in the RT group who received salvage TMZ after progression.

f The Eastern Cooperative Oncology Group (ECOG) performance status.

∧ patients aged <70 yrs who were with unfavorable KPS were also included.

# statistically significant imbalance in prognostic characteristics between the groups.

* six patients received other chemotherapeutics (e.g., carmustine, irinotecan).

The definition of elderly patients varied between the 21 NRSs. Eleven studies defined the term “elderly” as an age over 65 years [5], [15]–[16], [18], [20]–[21], [24], [26], [29]–[30], [34] and five studies defined “elderly” as over 70 years [19], [22], [31]–[33]. One study reported a cohort with all patients aged over 80 years [23]. Four NRSs with patients <65 years were also included because most of their patients were eligible for our study [17], [25], [27]–[28].

Variations of interventions of interest were common between the NRSs. Most patients in experimental groups were treated according to the EORTC 26981 trial [6], whereas three NRSs used adjuvant TMZ only after the completion of RT [16], [21], [28]. Abbreviated RT was also used either in combination with TMZ or alone as a control treatment. In addition, a small number of patients from three NRSs received alternative treatment options (e.g., surgery only, upfront TMZ other chemotherapeutics)[25]–[26], [29].

Because several outcomes of interest could not be extracted from the included NRSs, 16 studies [5], [16]–[19], [21]–[30], [32] were finally eligible for OS analysis, and nine were eligible for PFS analysis [16], [18], [22]–[24], [26]–[28], [32]. An assessment of the risk of bias showed no apparent difference across the NRSs in most domains of biases, except for selection bias. The risk of bias in the eligible NRSs was thus judged based on those variations, as described in Table S2. For OS analysis, eight NRSs were considered to be of lower risk [16], [19], [22], [25]–[26], [28]–[30], four were of higher risk [18], [23]–[24], [27], and four were of unclear risk [5], [17], [21], [32]. For PFS analysis, four NRSs were considered to be of lower risk [16], [22], [26], [28], four were of higher risk [18], [23]–[24], [27], and one was of unclear risk [32].

### Survival Data

**Figure 2:**
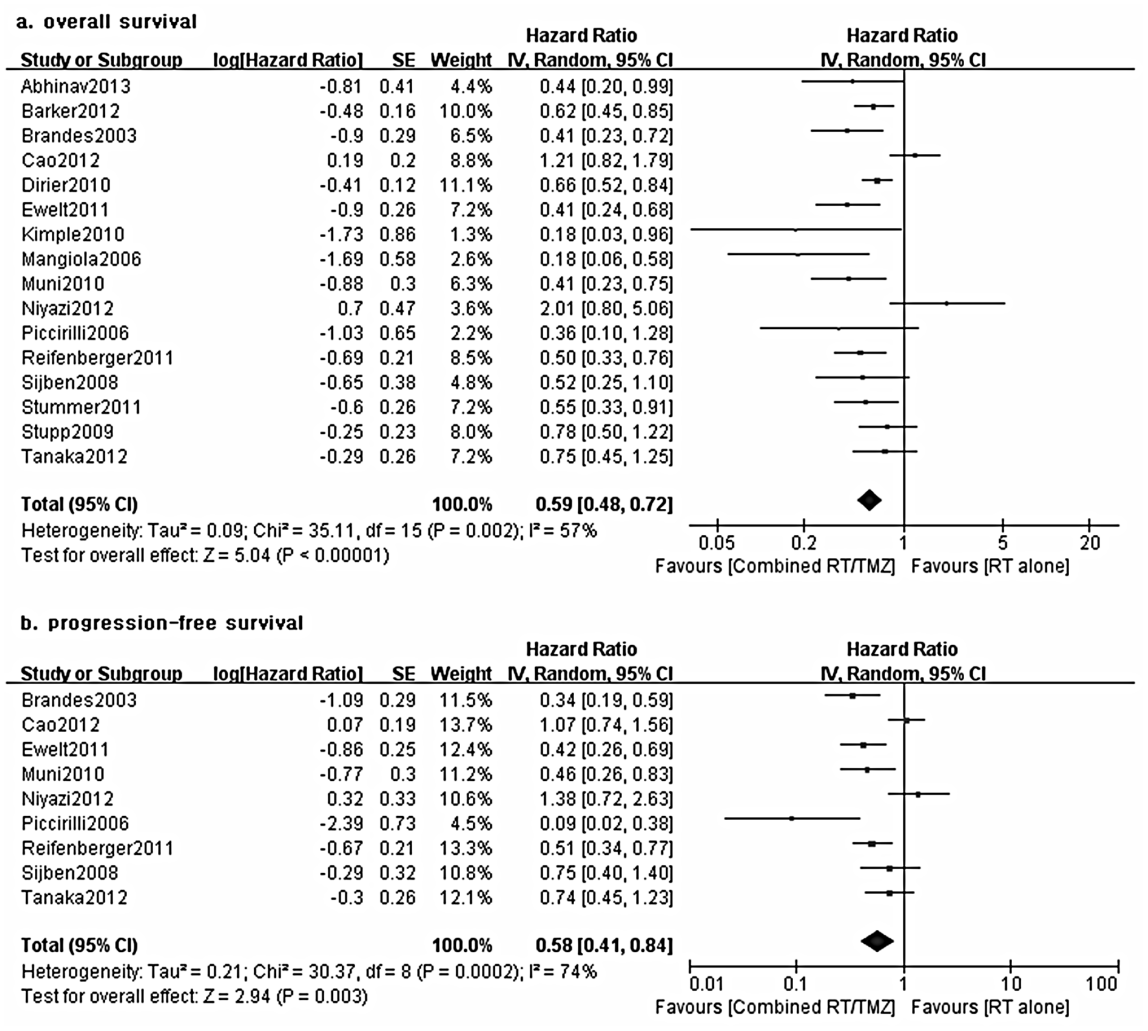
Forest plot of comparison: Combined RT/TMZ versus RT alone, outcome: a) overall survival; b) progression-free survival.

By using a random-effect model, a meta-analysis for OS demonstrated a 41% reduction in the risk of death with combined RT/TMZ (16 NRSs, 1492 elderly patients; HR 0.59, 95% CI 0.48–0.72, *P*<0.00001; test for heterogeneity: Chi^2^ = 35.11, *P* = 0.002, I^2^ = 57%; Fig. 2a). The superiority of combined treatment was also confirmed by a random-effect meta-analysis for PFS (9 NRSs, 399 elderly patients; HR 0.58, 95% CI 0.41–0.84, P = 0.003; test for heterogeneity: Chi^2^ = 30.37, *P* = 0.0002; I^2^ = 74%; Fig. 2b). Moreover, the sensitivity analyses incorporating only the studies with a lower risk of bias yielded consistent survival benefits for combined therapy (Table 2). However, it should be noted that the aggregate estimate of the survival rates of the RT groups in our study was not much inferior to the data in the EORTC26981 trial with younger patients, which indicated a possibility of highly selected patients (present study vs. the EORTC 26981 trial: PFS at 6 months, 39% vs. 36%; PFS at 12 months, 9% vs. 9%; OS at 12 months, 34% vs. 51%; OS at 24 months, 8% vs. 10%).

**Table 2:** The results of additional analyses.

Additional analysis	HR [95% CI]	*P*_Z-test_	I^2^ statistic
**Primary analysis**			
OS	0.59 [0.48, 0.72]	0.00	57%
PFS	0.58 [0.41, 0.84]	0.00	74%
**Duval and Tweedie’s ** ***trim and fill*** *			
Adjusted OS	0.65 [0.53, 0.80]	0.00	–
Adjusted PFS	0.64 [0.44, 0.93]	0.02	–
**Subgroup analysis**			
** OS**			
* 1. Study design features* (*P*_subgroup difference_ = 0.43)			
Prospective study	0.53 [0.42, 0.67]	0.00	9%
Retrospective study	0.62 [0.46, 0.83]	0.00	64%
* 2. Different RT schedules* (*P*_subgroup difference_ = 0.90)			
Short course RT	0.72 [0.25, 2.07]	0.55	89%
Standard RT	0.57 [0.44, 0.74]	0.00	51%
Unclear or mixed RT	0.56 [0.44, 0.71]	0.00	0%
*3. Different TMZ schedules* (*P*_subgroup difference_ = 0.02)			
Sequential TMZ	0.37 [0.26, 0.55]	0.00	0%
Concurrent TMZ	0.73 [0.55, 0.95]	0.02	52%
Unclear or either TMZ	0.59 [0.38, 0.72]	0.00	45%#
**Sensitivity analysis**			
** OS**			
Studies with lower risk of bias	0.57 [0.43, 0.77]	0.00	47%#
Studies with lower and unclear risk of bias	0.58 [0.47, 0.71]	0.00	46%#
Studies with all patients aged ≥65 years	0.55 [0.44, 0.70]	0.00	46%#
Excluding studies with other treatment options	0.58 [0.45, 0.75]	0.00	64%
** PFS**			
Studies with lower risk of bias	0.62 [0.35, 1.10]	0.11	74%
Studies with lower and unclear risk of bias	0.59 [0.39, 0.90]	0.02	67%
Studies with all patients aged ≥65 years	0.54 [0.36, 0.81]	0.00	70%

OS: overall survival; PFS: progression-free survival; TMZ: temozolomide; RT: radiotherapy;

* To remove the slight asymmetry of the funnel plots, four studies were trimmed for OS analysis and one for PFS analysis.

# Random-effect model was used despite I^2^ statistic <50% because other apparent heterogeneities were observed (e.g., variations in interventions of interest, different study designs).

### Toxicity Data

Thirteen included NRSs [15]–[17], [19], [21], [23]–[28], [30]–[31] (Table 1), along with a substantial number of uncontrolled studies [5], [35]–[42] (Table 3), had incorporated a safety analysis in which elderly patients were able to tolerate combined treatment. However, certain toxicities, and especially hematological adverse events (HAEs), were commonly observed.

**Table 3:** A summary of uncontrolled studies on the efficacy and safety of chemoradiation with TMZ for GBMs in the elderly.

Study reference	Cutoffage	Period ofdiagnosis	No. ofpatients	Therapy	Medianage (yrs)	median KPS(range)	Male%	Sx%	CR%	Median OS (mths,95% CI)	Median PFS (mths,95% CI)	Patients at leastone G3–4 HAE
**Prospective studies**												
*Short course RT*												
Floyd 2012 [35]	≥65	2007–2010	20	RT+SRS+concurrent TMZ	75.4^b^	70 (70–90)	45	65	50	13 (nr)	11 (nr)	–
Minniti 2009 [36]	≥70	2002–2006	43	RT +sequential TMZ	73	70 (60–90)	49	–	16	9.3 (7.5–11.1)	6.3 (4.8–7.8)	12 in 43
Minniti 2012 [37]	≥70	2005–2010	71	RT +concurrent TMZ	73	70 (60–100)	51	87	15	12.4 (9.9–15.0)	6 (4.1–8.2)	–
*Standard RT*												
Balducci 2012 [38]	≥65	2001–2008	56	RT +concurrent TMZ	69	nr (70–100)	54	100	41	14 (nr)	11 (nr)	–
Brandes 2009 [39]	≥65	2004–2007	58	RT +concurrent TMZ	68	80 (70–100)	59	100	40	13.7 (10–17.3)	9.5 (8.6–10.5)	–
Fiorentino 2013 [40]	≥65	2001–2011	111	RT+ concurrent TMZ	71	nr (>70)	55	–	36	13 (nr)	10 (nr)	–
Minniti 2008 [41]	≥70	2001–2005	32	RT +concurrent TMZ	73.6	80 (70–100)	56	100	22	10.6 (8.6–12.6)	7 (5–9)	7 in 32
**Retrospective studies**												
*Short course RT*												
Reyngold 2012 [42]	≥60^a^	2004–2010	31	RT +concurrent TMZ	66	70 (nr)	52	42	13	11 (nr)	–	11 in 28
Weiss 2010 [43]	≥65	2004–2007	24	RT +concurrent TMZ	76.8	65 (30–80)	–	–	–	8.2 (nr)	–	–
*Standard RT*												
Combs 2008 [44]	≥65	1999–2007	43	RT +concurrent TMZ	67	–	67	67	27	11 (nr)	4 (nr)	4 in 43
Gerstein 2010 [45]	≥65	1999–2009	51	RT +concurrent TMZ	70	–	53	55	26	11.5 (6.7–16.3)	5.5 (3.7–7.3)	–
Hashem 2012 [46]	≥60	2004–2008	20	RT +either TMZ	65	–	70	50	10	12.1 (8.3–19.3)	–	–
Minniti 2011 [47]	≥70	2005–2009	83	RT +concurrent TMZ	73.2	80 (70–100)	54	–	23	12.8 (9.3–16.7)	7.5 (5.8–9.4)	23 in 83
Zachenhofer 2011 [48]	≥65	2006–2010	20	RT +concurrent TMZ	73	69^b^ (nr)	–	60	30	7.7 (nr)	–	–
*Mixed or unclear RT*												
Abdel-Karim 2012 [49]	≥60	–	30	RT +concurrent TMZ	66 c	70 (nr)^c^	63 c	–	–	11.8 (nr)	6 (nr)	–
			30	RT +concurrent TMZ						12.3 (nr)	6.5 (nr)	6 in 30
Fiorentino 2012 [50]	≥65	2005–2011	45	RT +concurrent TMZ	71	–	44	–	31	13 (nr)	8 (nr)	4 in 45
Fiorica 2009 [51]	≥65	2002–2007	42	RT +concurrent TMZ	71.3^b^	70 (60–100)	64	86	55	10.2 (9.0–13.4)	5.8 (nr)	–
Gerstner 2009 [52]	≥70	1998–2009	40	RT +concurrent TMZ or upfront TMZ	74^c^	80 (50–100)^c^	–	100^c^	38^c^	methylated: 16.3 (nr)unmethylated: 8.8 (nr)	methylated: 13.5 (nr)unmethylated: 8.2 (nr)	–
Mishima 2010 [53]	≥65	2006–2010	25	RT +concurrent TMZ	72	–	–	–	–	13.7 (nr)	8.3 (nr)	–
Laperriere 2010 [54]	≥65	2000–2007	30	RT +concurrent TMZ	–	–	–	–	–	–	–	6 in 30
Lee 2013 [55]	≥70	2006–2010	20	RT +concurrent TMZ	73	2(0–4)^d^	40	55	45	11.8 (8.4–14.8)	–	2 in 20
Saito 2011 [56]	≥65	2004–2010	57	RT +unclear TMZ	–	–	–	–	–	15.2 (13.1–18.3)	8.7 (6.0–11.7)	–

RT = radiotherapy; SRS = stereotactic radiosurgery; TMZ = temozolomide; either TMZ = either sequential or concurrent TMZ; Sx = surgery; CR = complete resection; PFS = progression free survival; OS = overall survival; CI = confidential interval; HAE = hematological adverse event; yrs = years; mths = months; nr = not reported.

a patients aged <60 years who had a KPS of 70 or less were also included.

b mean but not median.

c the whole cohort.

d The Eastern Cooperative Oncology Group (ECOG) performance status.

Toxicity reporting for the RT groups was very poor, with only nine cases of confusion/somnolence, two cranial compressions, two infections, one intracerebral hemorrhage and one corona radiata infarct in three NRSs [16], [26], [28]. Additionally, three HAEs occurred in the RT groups because of the use of corticosteroids and salvage TMZ upon progression. [15], [27].

**Figure 3:**
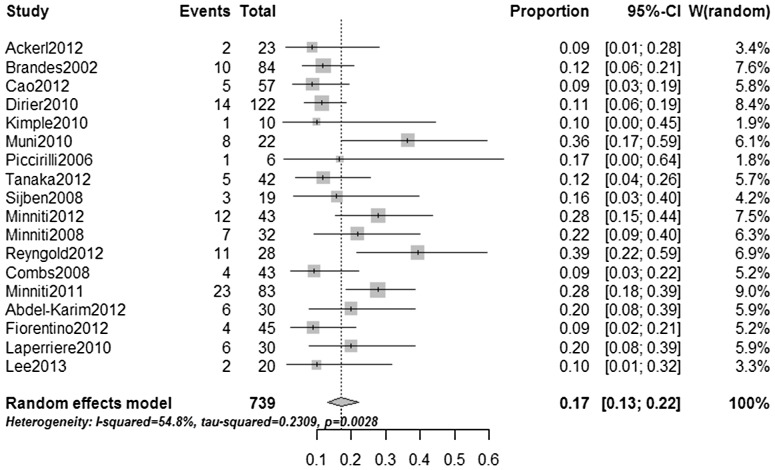
The aggregate estimate for the incidence of patients who experienced at least one grade 3–4 hematological adverse event in combined RT/TMZ groups.

Hematological toxicities were the major safety concern in elderly patients who were managed with combined treatment. The aggregate estimate of the percentage of patients who had at least one grade 3–4 HAE was calculated as 17%, with a 95% CI of 13–22% (Fig. 3). The percentage was similar to the value for younger patients (16%) [6]. Moreover, most of the toxicities were reversible, and no treatment-related deaths were reported. Elderly patients in combined treatment groups were also reported to have numerous moderate non-hematological toxicities, including leukoencephalopathy, cerebral compression, memory loss, skin rash, constipation, depression, fatigue, nausea/vomiting, transaminase elevation, stomatitis and infections.

### HRQOL Data

None of the included NRSs assessed the patients’ HRQOL using a validated scale (e.g., QLQ-C30, FACT-Br). Instead, four studies reported changes in the pre- and post-adjuvant treatment KPSs of their patients, which were comparable between the groups [21], [26], [28], [30].

### Additional Analysis

**Figure 4:**
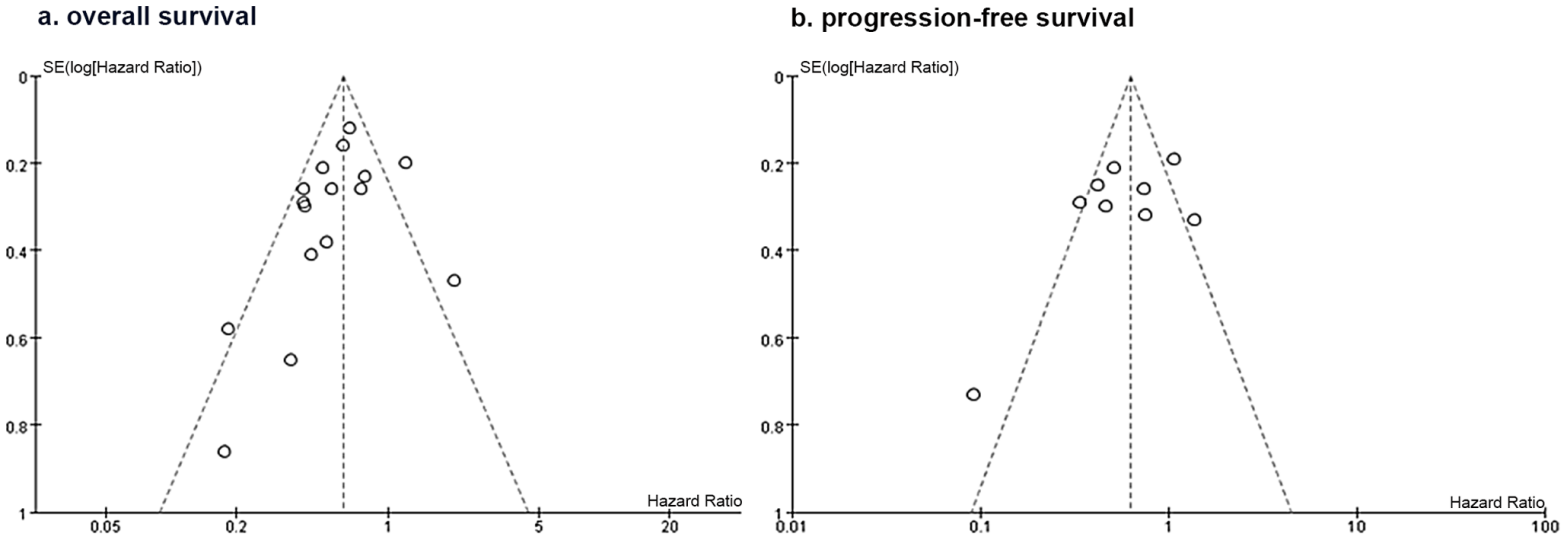
Funnel plot of comparison: Combined RT/TMZ versus RT alone, outcome: a) overall survival; b) progression-free survival.

The visual impression indicated a possibility of publication bias due to the slight asymmetry of the funnel plots for OS and PFS (Fig. 4). Egger’s test [12] did not yield statistically significant results, which should be carefully interpreted due to the low power of the test (OS: *P*_Egger’s test_ = 0.17; PFS: *P*_Egger’s test_ = 0.20). Duval and Tweedie’s *trim and fill* [13] was thus used to adjust the pooled statistics (Table 2).

Subgroup analyses did not identify any apparent heterogeneity by different study design features and different RT doses, whereas differences by two chemotherapy schedules (sequential vs. concurrent RT/TMZ) were found to be statistically significant (*P_subgroup difference_* = 0.02). Additionally, a meta-regression analysis of the restricted maximum likelihood approach did not yield any statistically significant variations in estimated statistics by variables such as median age, the percentage of patients with a KPS ≥70, the percentage of male participants and sample size, with the exception of the percentage of patients who underwent surgery. This variable seemed to be positively correlated with the survival benefits conferred by combined treatment (*P_meta-regression_* = 0.001). Overall, these observed variations might account for the between-study heterogeneity to a certain extent.

Sensitivity analysis did not show any apparent variations in pooled HRs for OS and PFS, supporting the robustness of the primary findings.

The results of additional analyses are presented in Table 2.

## Discussion

Elderly patients tend to be undertreated by their physicians due to innate bias that associates older age with inferior outcomes and severe toxicity. Limited data from clinical studies that validate the bias further lead to a vicious cycle of care that is rarely evidence based. This meta-analysis of NRSs indicated a clear survival advantage for combined RT/TMZ compared with RT alone among elderly GBM patients. Benefits were also highlighted in a substantial number of uncontrolled studies (Table 3) [35]–[56]. Regarding safety, combined treatment was considered to be safe for the evaluable older patients, but a notable rate of hematological toxicities was observed during treatment. Although appealing, the presented findings needed future randomized trials for validation.

The apparent benefits should be conservatively interpreted because these benefits might result from the highly selected older cohort in the included studies. In 14 NRSs [16]–[19], [21]–[30] with surgery information, 76% of patients had surgical resection, which was more frequent than in population-based studies (47–61%) [2]–[3]. The extent of resection was an independent prognostic factor in GBM patients. Much of the data favored a causal influence of extensive resection on prolonged survival [57]–[58]. Furthermore, an ancillary analysis of the EORTC 26981 trial suggested the possibility of a synergistic effect of resection on the efficacy of adjuvant radiochemotherapy [59]. Therefore, overall better surgical conditions may confer a systematic bias toward a larger benefit of combined RT/TMZ in elderly patients. More interestingly, we found that surgical conditions were the poorest in the two NRSs [22], [27] favoring treatment with RT alone, with only 42% and 11% patients in the combined treatment groups receiving surgery. This finding suggested that combined radiochemotherapy might not be beneficial for elderly patients who have undergone only a biopsy.

Overall favorable KPS scoring might also account for the apparent benefits observed in the evaluable cohort. In this meta-analysis, 84% of patients had a KPS ≥70 in eight NRSs [17]–[19], [22]–[25], [28], and 73% had a KPS ≥80 in seven NRSs [19], [22]–[23], [25], [27]–[28], [30]. The efficacy of the aggressive combination of RT/TMZ, however, was less justified in those patients with a lower KPS. In a study by Niyazi et al [22], the survival of patients with a KPS of 70 was shorter with combined RT/TMZ than with RT alone (*P* = 0.027) whereas survival became comparable when patients with a KPS of 80 were analyzed. Kimple et al [19] reported a similar result. It was hypothesized that a lower KPS is associated with poorer physical condition, a lower possibility of surgery and decreased tolerance to aggressive treatments. Therefore, the observed benefits might be largely confined to the older cohort with a favorable KPS.

MGMT is a DNA repair protein that protects GBM tumor cells from alkylating agents [60]. The promoter status of MGMT has been demonstrated to be a useful biomarker in younger GBM patients. The clinical value however, is less validated in the elderly population. In the present study, only four NRSs [23]–[24], [29], [32] tested MGMT promoter status. One study [32] reported that methylated tumors were associated with longer OS than unmethylated tumors among patients with combined treatment but not RT alone, whereas the other three studies [23]–[24], [29] did not yield a statistically significant association, which may be due to small sample size. Given the possibility of the high impact of this biomarker, especially in older GBM patients, the limited data on MGMT promoter status in the included NRSs disallowed a full interpretation of the presented findings. Recently, two phase 3 trials have highlighted the predictive value of this biomarker when selecting elderly patients for optimal individualized treatment [61]–[62]. In these studies, survival seemed to be better with upfront TMZ than with RT alone among patients with methylated tumors, and the opposite was true for those patients with unmethylated tumors. A comprehensive MGMT promoter methylation analysis is planned in the ongoing NCIC CE.6/EORTC 26062/22061 trial [14], and we await more results that define the clinical relevance of this biomarker in the elderly population.

Biases derived from study design and performance can also skew our results [8]. Assessing the risk of bias using standard evaluation tools is a reliable way to control the biases in a meta-analysis. Fewer tools, however, were justified for NRS assessment [8]. One potential useful tool is the NOS [9]. The scale-based NOS evaluates the risk of bias of NRSs by calculating a summary score of stars, which inevitably involves assigning “weights” to different items. In fact, it is difficult to justify the assigned weights. Moreover, the tool cannot cover all possible sources of biases in NRSs. [8] Therefore, to provide a more reliable validation of our review, we decide to modify the NOS by 1) completing the missing domains of biases; 2) assigning specific responses, such as “yes”, “no” or “unclear”, instead of stars, to each item; and 3) ranking the risk of bias of each study according to its overall responses, rather than the scoring of stars (see Table S1–S2).

To avoid worrisome heterogeneity, we conducted both a subgroup analysis and a meta-regression to explain variations. Differing proportions of patients who received surgical resection were demonstrated to be a possible source of between-study heterogeneity. Additionally, apparent subgroup heterogeneity was observed, such that sequential RT/TMZ appeared to be more beneficial than concurrent RT/TMZ in elderly patients. However, the result was not supported by other studies and may be complicated by other uncontrolled potential variables associated with the outcomes of interest, such as the risk of bias of each study, individual prognostic factors and combined RT schedules. Moreover, it should be noted that a subgroup analysis always has low statistical power to justify its results in real situations. Overall, the superiority of sequential RT/TMZ was not justified, and primary studies are warranted to determine optimal adjuvant chemotherapy schedules.

### Limitations of the Study

#### Potential risk of bias in NRSs

Potential biases are inherently greater in NRSs compared with randomized trials [8]. Selection bias is the major concern in most NRSs, in which an imbalance in prognostic factors associated with outcomes of interest is likely to occur between different groups. Additionally, poor performance and reporting of study protocols, assessment of prognostic factors and outcome measurement, which are common in NRSs, brought further uncertainty into the interpretation of the meta-analysis of NRSs.

#### Incomplete reporting of toxicity

Elderly patients are more liable to treatment-related toxicity compared with younger patients [5]. An assessment of adverse effects is very important in evaluating the superiority of an experimental intervention in older patients. However, “unbeneficial” outcomes were less likely to be assessed and reported in the included NRSs, and incomplete toxicity data prevented the full consideration of the use of combined treatment in the elderly cohort.

#### Paucity of data on HRQOL

HRQOL is a meaningful endpoint in a study of cancer patients, and especially elderly cancer patients [63]. However, only four included NRSs reported a less convincing HRQOL outcome, i.e., changes in pre- and post- treatment KPSs [21], [26], [28], [30]. Patient-reported HRQOL using a validated scale, which is believed to be the most reliable data [63], was not incorporated in any of the included NRSs.

## Conclusion

### Implications for Practice

The meta-analysis encouraged the use of aggressive combined treatment in elderly GBM patients by providing level 2a evidence that combined RT/TMZ yields a clear survival benefit in a selected older cohort with favorable prognostic factors (e.g., extensive resection, higher KPS) compared with RT alone. Toxicities appeared to be more frequent but acceptable. Patient selection is a major consideration when combined treatment is planned.

### Implications for Research

The NCIC CE.6/EORTC 26062/22061 trial is ongoing and will justify drawing definitive conclusion [14]. Future studies are warranted to determine the optimal combination of TMZ and RT and to verify the clinical relevance of MGMT promoter methylation in older patients. Moreover, given that elderly GBM patients are heterogeneous, with various responses to therapies, a combination of performance status, the extent of resection, comorbidity, clinically relevant biomarkers (e.g., MGMT promoter status) and chronological age should be investigated in future trials to help stratify older patients for optimal individualized treatment, along with a standardized assessment of toxicity and HRQOL.

## Supporting Information

Table S1 **Criteria for judgment risk of bias in the modified domain-based evaluation.**
(DOC)

Table S2 **Assessment of risk of bias of included nonrandomized studies.**
(DOC)

Checklist S1 **PRISMA Checklist.**
(DOC)
