# Supplementary material for: Radiotherapy Plus Concurrent or Sequential Temozolomide for Glioblastoma in the Elderly: A Meta-Analysis
Source: PLoS One. 2013 Sep 24;8(9):e74242. doi: 10.1371/journal.pone.0074242 (PMC3782499; doi:10.1371/journal.pone.0074242)
Supplement: Table S2 — Assessment of risk of bias of included nonrandomized studies. (DOC) [file pone.0074242.s002.doc]

Supplemental material Table S2: Assessment of risk of bias of included nonrandomized studies

| ***Domains*** | Brandes | Barker | Kimple | Niyazi | Sijben | Stummer | Stupp | Cao | Muni |  |
| --- | --- | --- | --- | --- | --- | --- | --- | --- | --- | --- |
|  | 2003[16] | 2012[30] | 2010[19] | 2012[22] | 2008[24] | 2011[25] | 2009[5] | 2012[27] | 2010[28] |  |
| ***Selection bias*** |  |  |  |  |  |  |  |  |  |  |
| *1. Selection* |  |  |  |  |  |  |  |  |  |  |
| 1.1 Allocation of participants: any criteria reported? | *Yes* | *Yes* | *Yes* | *Yes* | *Yes* | *Yes* | *Yes* | *Yes* | *No* |  |
| 1.2 How representative was the groups of RT alone in comparison with the general elderly patients with GBMs? | *Yes* | *Yes* | *Yes* | *Yes* | *Yes* | *Yes* | *Yes* | *Yes* | *Yes* |  |
| 1.3 How representative was the groups of combined RT/TMZ in comparison with the general elderly patients with GBMs? | *Yes* | *Yes* | *Yes* | *Yes* | *Yes* | *Yes* | *Yes* | *Yes* | *Yes* |  |
| *2. Comparability* |  |  |  |  |  |  |  |  |  |  |
| 2.1 Group comparable for: *1)* age; *2)* extent of resection; *3)* performance status (e.g. KPS); *4)* gender | *Yes* | *Yes* | *Yes* | *Yes* | *No* | *No* | *Unclear* | *No* | *Yes* |  |
| 2.2 Group comparable for: *5)* tumor location or numbers; *6)* neurological status; *7)* the MGMT promoter status; *8)* comorbidities | *Unclear* | *Unclear* | *Unclear* | *Unclear* | *Unclear* | *Unclear* | *Unclear* | *Unclear* | *Unclear* |  |
| 2.3 Control for confounding (OS) ^#^ | *No* | *Yes* | *No* | *Yes* | *No* | *Yes* | *Unclear* | *No* | *No* |  |
| 2.4 Control for confounding (PFS) ^#^ | *No* | *Unclear* | *Unclear* | *No* | *No* | *Unclear* | *Unclear* | *No* | *No* |  |
| ***Performance bias*** |  |  |  |  |  |  |  |  |  |  |
| 3. Blinding of participants and personnel* | *Yes* | *Yes* | *Yes* | *Yes* | *Yes* | *Yes* | *Yes* | *Yes* | *Yes* |  |
| 4. Exposure of interventions | *Yes* | *Yes* | *Yes* | *Yes* | *Yes* | *Yes* | *Yes* | *Yes* | *Yes* |  |
| ***Detection bias*** |  |  |  |  |  |  |  |  |  |  |
| 5. Blinding of outcome assessment* | *Yes* | *Yes* | *Yes* | *Yes* | *Yes* | *Yes* | *Yes* | *Yes* | *Yes* |  |
| 6. Ascertainment of outcome data | *Yes* | *Yes* | *Yes* | *Yes* | *Yes* | *Yes* | *Yes* | *Yes* | *Yes* |  |
| ***Attrition bias*** |  |  |  |  |  |  |  |  |  |  |
| 7.1 Adequacy of outcome data (OS) | *Yes* | *Yes* | *Yes* | *Yes* | *Yes* | *Yes* | *Yes* | *Yes* | *Yes* |  |
| 7.2 Adequacy of outcome data (PFS) | *Yes* | *Unclear* | *Unclear* | *Yes* | *Yes* | *Unclear* | *Unclear* | *Yes* | *Yes* |  |
| ***Reporting bias*** |  |  |  |  |  |  |  |  |  |  |
| 8. Selective outcome reporting | *Unclear* | *Unclear* | *Unclear* | *Unclear* | *Unclear* | *Unclear* | *Unclear* | *Unclear* | *Unclear* |  |

| ***Domains*** | Abhinav | Dirier | Ewelt | Mangiola | Piccirilli | Reifenberger | Tanaka |
| --- | --- | --- | --- | --- | --- | --- | --- |
|  | 2013[29] | 2010[17] | 2011[18] | 2006[21] | 2006[23] | 2011[32] | 2012[26] |
| ***Selection bias*** |  |  |  |  |  |  |  |
| *1. Selection* |  |  |  |  |  |  |  |
| 1.1 Allocation of participants: any criteria reported? | *Yes* | *Yes* | *Yes* | *Yes* | *Yes* | *Yes* | *Yes* |
| 1.2 How representative was the groups of RT alone in comparison with the general elderly patients with GBMs? | *Yes* | *Yes* | *Yes* | *Yes* | *Yes* | *Yes* | *Yes* |
| 1.3 How representative was the groups of combined RT/TMZ in comparison with the general elderly patients with GBMs? | *Yes* | *Yes* | *Yes* | *Yes* | *Yes* | *Yes* | *Yes* |
| *2. Comparability* |  |  |  |  |  |  |  |
| 2.1 Group comparable for: *1)* age; *2)* extent of resection; *3)* performance status (e.g. KPS); *4)* gender | *Unclear* | *Unclear* | *No* | *Unclear* | *No* | *Unclear* | *No* |
| 2.2 Group comparable for: *5)* tumor location or numbers; *6)* neurological status; *7)* the MGMT promoter status; *8)* comorbidities | *Unclear* | *Unclear* | *Unclear* | *Unclear* | *Unclear* | *Unclear* | *Unclear* |
| 2.3 Control for confounding (OS)^#^ | *Yes* | *No* | *No* | *No* | *No* | *No* | *Yes* |
| 2.4 Control for confounding (PFS)^#^ | *Unclear* | *Unclear* | *No* | *Unclear* | *No* | *No* | *Yes* |
| ***Performance bias*** |  |  |  |  |  |  |  |
| 3. Blinding of participants and personnel* | *Yes* | *Yes* | *Yes* | *Yes* | *Yes* | *Yes* | *Yes* |
| 4. Exposure of interventions | *Unclear* | *Unclear* | *Yes* | *Yes* | *Yes* | *Yes* | *Yes* |
| ***Detection bias*** |  |  |  |  |  |  |  |
| 5. Blinding of outcome assessment* | *Yes* | *Yes* | *Yes* | *Yes* | *Yes* | *Yes* | *Yes* |
| 6. Ascertainment of outcome data | *Unclear* | *Unclear* | *Yes* | *Yes* | *Yes* | *Yes* | *Yes* |
| ***Attrition bias*** |  |  |  |  |  |  |  |
| 7.1 Adequacy of outcome data (OS) | *Yes* | *Yes* | *Yes* | *Yes* | *Yes* | *Yes* | *Yes* |
| 7.2 Adequacy of outcome data (PFS) | *Unclear* | *Unclear* | *Yes* | *Unclear* | *Yes* | *Yes* | *Yes* |
| ***Reporting bias*** |  |  |  |  |  |  |  |
| 8. Selective outcome reporting | *Unclear* | *Unclear* | *Unclear* | *Unclear* | *Unclear* | *Unclear* | *Unclear* |

* Yes was assigned despite that blinding was not conducted because the reviewers authors judged that OS and PFS were unlikely to be influenced by lack of blinding

^#^ The study was considered of lower risk in selection bias if appropriate methods were used to control for the potential confounders (e.g., imbalanced prognostic factors.)
